# I/D Polymorphism Gene ACE and Risk of Preeclampsia in Women with Gestational Diabetes Mellitus

**DOI:** 10.1155/2020/8875230

**Published:** 2020-12-28

**Authors:** O. P. Dmitrenko, N. S. Karpova, M. K. Nurbekov, O. V. Papysheva

**Affiliations:** ^1^Federal State Budgetary Institution “Research Institute of Pathology and Pathophysiology”, 125315 Moscow, Russia; ^2^City Clinical Hospital № 29 named after N.E. Bauman, 123001 Moscow, Russia

## Abstract

Preeclampsia (PE) and gestational diabetes mellitus (GDM) are the most common complications of pregnancy, which result in adverse outcomes for the mother and the fetus. GDM is regarded as a separate independent risk factor for PE development, as evidenced by a higher preeclampsia rate in gestational diabetes mellitus than in the general population. The role the endothelial cell dysfunction plays is considered to be the most reasonable one in the origin of these diseases. The activity of plasma and tissue angiotensin converting enzyme (ACE) is believed to be genetically controlled. The available data suggests that increased ACE activity due to deletion (D)/insertion (I) in the 16th intron of ACE gene, which is called ACE gene I/D polymorphism, is associated with preeclampsia and varies depending on the studied population and the geography. We did not find any literature data that estimates the influence of ACE gene I/D polymorphism on PE rate in pregnant women with GDM. Therefore, the present study aimed to investigate a relationship between ACE gene I/D polymorphism and preeclampsia development in the case of GDM in the Russian population. The study used the genomic DNA derived by phenol-chloroform extraction method from venous blood samples in 137 pregnant women, including samples of 74 women with GDM accompanied with PE and the blood samples of 63 women with GDM w/o preeclampsia. Genotyping of insertion/deletion in the I/D region (16 intron of АСЕ gene) was conducted by real-time PCR using the TaqMan competing probe technology. The particular features in the frequency array of alleles and genotypes of the ACE gen I/D polymorphism under review, as associated with preeclampsia development risk in pregnant women with GDM, were identified. The acquired data testify to the need to further study of ACE gene I/D region polymorphism association in a large patient sample taking into account the PE and GDM risk factors estimated in the clinical practice.

## 1. Introduction

Preeclampsia (PE) is a multi-system disorder that arises after gestation week 20 and is described by arterial hypertension, combined with proteinuria (≥0.3 g/l in daily urine), oedema and multi-organ dysfunction [1–3]. PE develops in 3%-8% pregnant women and is among the Top 5 reasons of maternal morbidity and mortality [4–8]. Data derived in different years points to a much greater preeclampsia rate in the case of gestational diabetes mellitus (7.3%) vs general in the population (4.5%) [9–12]. GDM is diabetes that is first diagnosed in the second or third trimester of pregnancy that is not clearly either preexisting type 1 or type 2 diabetes [13]. According to the International Diabetes Federation (IDF), gestation diabetes mellitus prevalence in 2019 decreased slightly, to 12.8%, varying depending on the population parameters, screening methods and diagnostic criteria: from 1% to 28% [14–17]. In Russia, GDM is a pregnancy complication in 2%-4% cases [18].

Even though the preeclampsia clinical manifestations and diagnostic criteria do not overlap with those of GDM, certain studies suggested that the same predisposing factors are typical of PE and GDM (mother's age of over 35 y.o., nulliparity, multiple pregnancy and increased pre-gestation BMI); however, GDM is a separate and independent risk factor of PE development [19–22].

Just as with GDM and with preeclampsia, the main pathophysiological criterion is the vascular endothelial dysfunction [23–26]. The renin-angiotensin-aldosterone system (RAS) is the main vascular tone regulator activation of which results in the blood pressure increase due to the growth in circulating blood and increase in activity of other vasoconstricting factors. The key role in the functioning of RAS belongs to the angiotensin-converting enzyme (ACE), which catalyzes the cleavage of angiotensin I Decapeptide to angiotensin II octapeptide, by releasing the terminal His-Leu, which improves the angiotensin vasoconstrictor activity. [27–29]. ACE has a significant effect on the production of angiotensin II not only in the circulating blood, but also on the synthesis and interaction of components of tissue RAS, including in the beta-cells of the Langerhans islets and the placenta [30–32]. Local RAS in the pancreas and the placenta are believed to be involved in physiological and pathophysiological processes in pregnancy [33].

The ACE gene is located on locus 17q23.3, in the DNA plus chain, which consists of 26 exons and 25 introns, and encodes an angiotensin-converting enzyme [34, 35]. The insertion/deletion (I/D) polymorphism in the 16th intron of ACE gene, which is described by presence or absence of the 264 bps Alu-repeats with a 15 bps poly-A-tail, as a partial explanation of inter-individual differences in ACE blood serum levels: the maximum one is detected in D/D genotype individuals, and twice as low level, in I/I type individuals [35–37].

I/D polymorphism of АСЕ gene is associated with ACE blood level and determines the onset of such diseases as coronary heart disease, left ventricular hypertrophy, arterial hypertension, stroke, diabetic nephropathy, type 2 diabetes mellitus, diabetic nephropathy, diabetic neuropathy, diabetic retinopathy and hypertensive retinopathy, according to different research [35, 38–46]. ACE gene D-alleles are believed to be a risk factor in these diseases.

The results of association studies of I/D polymorphism in the case of PE and GDM are inconsistent [47–58]. Some investigators reported that women residing in different regions had an association between ACE gene D-allele or DD genotype and a high preeclampsia risk [51–56], but others noted there was no such association with PE risk [57–58].

We did not find in the literature any data that estimates the influence of ACE gene I/D polymorphism on PE rate in pregnant women with GDM. Therefore, this study was aimed at investigating the relationship between I/D polymorphism of ACE gene and preeclampsia in the case of GDM.

## 2. Materials and Methods

The study involved DNA samples extracted from the venous blood of 137 GDM pregnant women, including those of 74 pregnant women with combined GDM and PE and those of 63 pregnant women with GDM not combined with preeclampsia, who were followed up and gave birth in 2018/2019 in the Maternity Department of the State Clinical Hospital No. 29 (N.E. Bauman Hospital) of the Healthcare Department of Moscow. All respondents were Russian speakers of indefinite ethnicity (because of the ethical standards of the local medical register) and enrolled in the study voluntarily. The study was approved by the Ethical Committee of the Research and Development Institute of General Pathology and Pathophysiology.

GDM and PE diagnosis were the study inclusion criteria. The absence of acute and chronic diseases at the exacerbation stage was the exclusion criterion. Pregnant women with autoimmune, nervous and mental diseases and with cancer of any localization were also excluded from the study.

GDM diagnosis was established based on the criteria of the Russian National Consensus and clinical guidelines “Gestational Diabetes Mellitus: Diagnosis, Treatment, Postpartum Follow-Up” [59].

Preeclampsia was diagnosed based on the clinical guidelines “Hypertensive Disorders in Pregnancy, Labor and Post-Partum. Pre-eclampsia. Eclampsia” [1].

DNA was extracted from venous blood with the standard phenol-chloroform extraction method of Maniatis et al. [60]. The blood cell element lysis was conducted with the Kunkel method [61]. The high-molecular DNA was desiccated at the ambient temperature and dissolved in ТЕ buffer, and the resulting DNA was stored at -20°С. All DNA extractions were performed by the single investigator only.

The extracted DNA quality and quantity was estimated by the 260/280 wavelength ratio when measuring DNA concentration in the NanoDrop 1000 spectrophotometer, in the two-chain DNA analysis mode, dsDNA-50. Defective samples were not included into further analysis, just as those with low DNA concentration.

The deletion/insertion in the I/D area (ACE gen 16th intron) was studied by real-time PCR (RT-PCR, q-PCR) using the primers we designed. The set of primers includes 2 forward primers: ACE-I/D-F1: 5'-GGAGACCACTCCCATCCTTTC-3' and ACE-I/D-F2: 5'-GCCTCAGCCTCCCAAG-3', and 1 reverse primer ACE-I/D-R1: 5'-ATGTGGCCATCACATTCGTCAGAT-3'. The primers were designed so that the ACE-I/D-F1 forward primer and the ACE-I/D-R1 reverse primer amplify the I/D area (ACE gen 16th intron) with insertion, while ACE-I/D-F2 and ACE-I/D-R1 primers, the I/D area (ACE gen 16th intron) with deletion. The ACE-I/D-R1 forward primer is annealed in the insertion area and creates a specific PCR product while the ACE-I/D-F2 primer, in a specific site that is formed in case of the Alu-repeat deletion only. To detect the amplification in real-time with subsequent analysis by the allele discrimination method, the specific Taq-man probes are used: ACE-I: 5'HEX-CCCGCCACTACGCCCGGCTAA-3'BHQ1 to detect an insertion and ACE-D: 5'FAM-GCTGCCTATACAGTCACTTTTATGTGGT-3'BHQ1 to detect a deletion. All primers and Taq-man probes were synthetically produced by Syntol LLC, Russia.

The reaction mixture for RT-PCR for one 15 *μ*l sample contained 20 ng DNA, 10 mM Tris–HCl (pH 8.3), 50 mM КСl, 3 mM MgCl2, 0.125 mM dNTP, 0,1 *μ*M ACE-I/D-F1, 0,2 *μ*M ACE-I/D-F2, 0,2 *μ*M ACE-I/D-R1, 0,2 *μ*M ACE-I, 0,2 *μ*M ACE-D, 0.4 *μ*M each of Taq-man probes, 0.25 units of act. TaqDNA-polymerase.

Amplification was carried out in the CFX 96 programmable amplifier (Bio-Rad, U.S.A.) with the subsequent thermocycling parameters: initial denaturation for 5 minutes at 95°С; then 40 cycles including denaturation at 95°С for 20 seconds, at 60°С for 30 seconds and at 25°С for 10 minutes; primer annealing and subsequent elongation at 72°С for 10 minutes, with subsequent fluorescence pickup. The obtained data was examined using the CFX Manager TM software (Bio-Rad).

The results were statistically processed using the Hardy-Weinberg equilibrium tests and the chi2 association test in DeFinetti application (freely available on the website of the Institute of Human Genetics (Germany), http://ihg.gsf.de/cgi-bin/hw/hwa1.pl.). The trend analysis was carried out using the general, dominant and recessive inheritance models. Differences were regarded as significant at *p* ≤ 0.05. The association force for the tested attributes was determined by the odds ratio (OR) with the confidence interval (CI) at 95% significance level. The anticipated risk factor was regarded as significant for pathology if OR adjusted by CI was greater than 1.

## 3. Results

The insertion/deletion test in the I/D area (ACE gen 16th intron) enabled to assess the allele frequency of the polymorphic loci genotypes in the studied gen. The distribution of genotype and allele frequencies in the I/D polymorphic locus of the ACE gen matched the one expected in the Hardy-Weinberg equilibrium, both for the pre-eclampsia group and for the control (no preeclampsia) group (Table 1).

The investigation into the distribution of genotype and allele frequencies in the study groups revealed that the D allele frequency came to 48.6% in the group of GDM PE+ pregnant women vs 37.3% in the group of GDM PE-. The frequency of DD homozygous genotype in the GDM PE+ group is higher than that in the GDM PE- group (24.3% vs 11.1%).The association analysis revealed the association between ACE gene I/D polymorphism and preeclampsia (Table 2). So, DD homozygous type in the general inheritance model is a genetic factor predisposing to this pregnancy complication in GDM women, increasing its risk by 2,96 (р =0,041).

## 4. Discussion

Preeclampsia and GDM are the most common pregnancy complications that result in an adverse outcome for the mother and the fetus. Despite numerous data on the reasons for these complications in the literature, the key drivers are still unknown; however, the role the endothelial cell dysfunction plays is considered to be the most probable cause of GDM and preeclampsia onset.

The research and clinical studies suggest that an array of genetic factors are involved in PE and GDM pathogenesis. So far, 100 polymorphic gene allelic variants, in particular, those regulating the endothelium, vascular system etc. functions, have been found to be associated with pre-eclampsia. Special focus was on ACE gen, one of the renin-angiotensin system encoding elements, because the Alu-repeat deletion of I/D polymorphism in ACE gen 16th intron leads to increased expression of the gen, which explains the inter-individual differences in angiotensin-converting-enzyme (ACE) blood levels. The available data suggests that persons with D/D genotype have the maximum ACE blood level and those with I/I genotype, twice as low level [55, 62].

The available reference data on the study of the ACE gen I/D polymorphism association with preeclampsia is inconsistent. Our study findings conform to some previous studies that pointed to the high frequency of DD genotype and/or D allele in preeclampsia. For instance, in the Asian region, ACE gene I/D polymorphism is associated with PE development risk in the Korean and Chinese populations [55, 63, 64]. Gürdöl F. et al. (2004), Bereketoğlu C. et al. (2012) reported high frequency of D allele in the Turkish population, Salimi S. et al. (2011), in the Iranian population [53, 56, 65]. According to several studies, high frequency of D allele is associated with the pre-eclampsia risk in the European population. For example, Mišković et al. (2008) found a significant association between D allele frequency and preeclampsia recurrence risk and preterm delivery before gestation week 34 [66]. The study of Mandò C et al. (2009) comprising 672 women, including 204 with pre-eclampsia pregnancy complication and 56 with gestation diabetes mellitus, suggested that D allele was much more common among women suffering from mild preeclampsia than it was in the control group [67]. Velloso et al. (2007) suggested that the DD genotype can be used as the marker of predisposition to preeclampsia in pregnant women in the Brazilian population [68].

On the other hand, some studies did not find any differences in the allocation of allele genotypes and frequencies and the association between DD genotype and preeclampsia [48, 58, 69–74]. In the association analysis, Radkov et al. (2012) established that the D heterozygous genotype and D allele were the genetic factors predisposing to preeclampsia in the Russian population residing in Central Russia, increasing the risk by the factor of 1.96 and 1.45, respectively [75]. When studying more than 1,500 polymorphisms (SNP) using high-density microchips, Glotov et al. (2014) identified several SNPs associated with the preeclampsia risk. These polymorphous sites enabled to identify 31 genes, including ACE, that can influence the disorder development. The further comparative analysis between Russian and Central European study groups did not identify any statistically significant differences in ACE gene allele and genotype frequencies [76].

The differences in the data derived from different studies on the association between ACE gene I/D polymorphism and preeclampsia are likely to be due to the size, population features and the ethnicity of the study groups and also depend on the analytical inheritance model selection. The meta-analysis conducted by Medica et al. (2006) on 10 studies with participation of 1,121 patients and 1,361 control group participants demonstrated the statistical significance in consideration of the I/D polymorphism of ACE gene 16th intron in the recessive model: the odds ratio came to 1.51 (95% CI: 1.17-1.94) [77]. Bereketoğlu C. et al. (2012) showed the connection between this polymorphism and pre-eclampsia in the dominant model [56].

The principal goal of this study was to investigate into the relationship between I/D polymorphism of АСЕ gene and the preeclampsia risk in pregnant women with gestational diabetes mellitus in the Russian population. Our study suggested that the frequency of the DD homozygous genotype of АСЕ gen varied between the groups, and D allele was more frequent in pre-eclampsia women than it was in PE- group. The relationship between this polymorphism and preeclampsia was found in the general inheritance model. We discovered a higher frequency of DD genotype in the preeclampsia group as compared with the control group. It is noteworthy that no studies of the association between ACE gene I/D polymorphism and preeclampsia development in GDM pregnant women have been conducted in the Russian population so far.

## 5. Conclusions

Our study revealed a statistically significant association between the DD homozygous type of ACE gene I/D polymorphism and preeclampsia in GDM women in the general inheritance model. A small size of the study groups is the main limitation of this study. Nonetheless, the data we received point to the need for further study of the association between ACE gene I/D polymorphism in a large patient sampling, with the parallel trial of polymorphism in other genes and taking into account PE and GDM risk factors estimated in the clinical practice (mother's age, BMI, glucose and glycated hemoglobin level etc.), which can also affect the development of these disorders. In the future, it will enable to use this genetic marker as the criterion in assessing the individual forecast of preeclampsia development in GDM pregnant women, which will enable to take efficient preventive efforts for timely correction and improvement of the pregnancy outcome.

## Figures and Tables

**Table 1:** Distribution of alleles and genotypes of ACE gene I/D polymorphism in GDM pregnant women in PE+ and PE- groups.

Genotypes and alleles	PE+, *n*=74	PE-, *n*=63
II	20 (27.0%)	23 (36.5%)
ID	36 (48.7%)	33 (52.4%)
DD	18 (24.3%)	7 (11.1%)
I	76 (51.4%)	79 (62.7%)
D	72 (48.6%)	47 (37.3%)

II: insertion genotype; ID: insertion/deletion genotype; DD: deletion genotype; I: insertion allele; D: deletion allele. For example, in the PE+ group II indicates 20 (27%) is the genotype number, and in the parentheses, it indicates the percentage of the alleles frequency.

**Table 2:** Association of ACE gene I/D polymorphism genotypes with PE in GDM pregnant women.

Model of inheritance	Genotypes	PE+, n =74	PE-, n =63	OR (95% of CI)	chi2	P
Codominant	II	20	23	0,338 (0,117-0,975)	4,17	0,041∗
	ID	36	33	1,255 (0,585-2,691)	0,34	0,559
	DD	18	7	2.957 (1,026-8,526)	4,17	0,041∗

Dominant	II/ID + DD	20/54	23/40	1,552 (0,752-3,207)	1,42	0,233

Recessive	II + ID/DD	56/18	56/7	0,389 (0,151-1,004)	3,98	0,046∗

Control (PE-) versus PE+; OR: odds ratio; CI: confidence interval; chi2: chi-square distribution; *p*: *p* value definition. ^∗^*p* ≤ 0.05.

## Data Availability

Genotyping data used to support the findings of this study is available upon request to the authors of the article.

## Abbreviations

PE: Preeclampsia
GDM: Gestational diabetes mellitus
IDF: International diabetes federation
BMI: Body mass index
RAS: The renin-angiotensin-aldosterone system
ACE: The angiotensin-converting enzyme.
